# In Vivo Confocal Microscopy in Scarring Trachoma

**DOI:** 10.1016/j.ophtha.2011.04.014

**Published:** 2011-11

**Authors:** Victor H. Hu, Helen A. Weiss, Patrick Massae, Paul Courtright, William Makupa, David C.W. Mabey, Robin L. Bailey, Matthew J. Burton

**Affiliations:** 1 International Centre for Eye Health, Faculty of Infectious and Tropical Diseases, London School of Hygiene and Tropical Medicine, London, United Kingdom; 2 Kilimanjaro Centre for Community Ophthalmology, Moshi, Tanzania; 3 MRC Tropical Epidemiology Group, London School of Hygiene and Tropical Medicine, London, United Kingdom; 4 Clinical Research Department, Faculty of Infectious and Tropical Diseases, London School of Hygiene and Tropical Medicine, London, United Kingdom

## Abstract

**Objective:**

To characterize the tissue and cellular changes found in trachomatous scarring (TS) and inflammation using in vivo confocal microscopy (IVCM).

**Design:**

Two complimentary case-control studies.

**Participants:**

The first study included 363 cases with TS (without trichiasis), of whom 328 had IVCM assessment, and 363 control subjects, of whom 319 had IVCM assessment. The second study included 34 cases with trachomatous trichiasis (TT), of whom 28 had IVCM assessment, and 33 control subjects, of whom 26 had IVCM assessment.

**Methods:**

All participants were examined with ×2.5 loupes. The IVCM examination of the upper tarsal conjunctiva was carried out with a Heidelberg Retina Tomograph 3 with the Rostock Cornea Module (Heidelberg Engineering GmbH, Dossenheim, Germany).

**Main Outcome Measures:**

The IVCM images were graded in a masked manner using a previously published grading system evaluating the inflammatory infiltrate density; the presence or absence of dendritiform cells (DCs), tissue edema, and papillae; and the level of subepithelial connective tissue organization.

**Results:**

Subjects with clinical scarring had a characteristic appearance on IVCM of well-defined bands and sheets of scar tissue visible. Similar changes were also seen in some clinically normal subjects consistent with subclinical scarring. Scarred subjects had more DCs and an elevated inflammatory infiltrate, even after adjusting for other factors, including the level of clinical inflammation. Cellular activity was usually seen only in or just below the epithelium, rarely being seen deeper than 30 μm from the surface. The presence of tissue edema was strongly associated with the level of clinical inflammation.

**Conclusions:**

In vivo confocal microscopy can be quantitatively used to study inflammatory and scarring changes in the conjunctiva. Dendritic cells seem to be closely associated with the scarring process in trachoma and are likely to be an important target in antifibrotic therapies or the development of a chlamydial vaccine. The increased number of inflammatory cells seen in scarred subjects is consistent with the immunopathologic nature of the disease. The localization of cellular activity close to the conjunctival surface supports the view that the epithelium plays a central role in the pathogenesis of trachoma.

**Financial Disclosure(s):**

The author(s) have no proprietary or commercial interest in any materials discussed in this article.

Trachoma is a chronic, cicatrizing keratoconjunctivitis and is the most common infectious cause of blindness globally.^1^ Trachoma is caused by infection with *Chlamydia trachomatis*, serovars A–C. In endemic regions, chlamydial infection is most frequently found in children, who develop a follicular and papillary conjunctivitis and sometimes corneal pannus (vascular infiltration). After repeated episodes of infection and inflammation, the scarring complications of trachoma develop: conjunctival scarring, entropion, trichiasis, and corneal opacity. Although there has been an encouraging downward trend in the number of people with active trachoma over the last 3 decades, this disease remains a significant public health problem in >50 countries.^2^

Despite numerous studies on the pathogenesis of scarring trachoma, the factors driving the late-stage scarring process and the immunofibrogenic responses involved remain to be elucidated.^3^ In vivo confocal microscopy (IVCM) is a new technique that provides high-resolution images of the ocular surface down to the cellular level. It has been used to study a wide range of infectious, inflammatory, and metabolic disease processes affecting the ocular surface.^4–9^ We have previously reported a description of the main features of trachoma seen with IVCM and a new grading system for the quantitative assessment of these images.^10^ With the use of IVCM, one can assess the type and density of inflammatory cell infiltrates and the morphology and distribution of fibrotic tissue within the tarsal conjunctiva. In vivo confocal microscopy is noninvasive, has no adverse effects, and can be repeated in the same individual.

To better understand the disease process in scarring trachoma, we conducted 2 case-controls studies looking at the IVCM findings in individuals with mild-moderate scarring (trachomatous scarring [TS]) and severe scarring (trachomatous trichiasis [TT]) compared with normal controls. It is anticipated that this technology can be used to support the detailed assessment of progressive scarring in the study of both current and future interventions in trachoma control.

## Patients and Methods

### Ethical Permission and Subject Recruitment

This study was approved by ethics committees in Tanzania and the United Kingdom; written, informed consent was obtained from each subject. The first study, on trachomatous scarring, was conducted in the Siha District of the Kilimanjaro Region of Northern Tanzania, in what was historically a single village. Two years before the study, this village was subdivided into 3 administrative units that form a single continuous geographic entity. Previous surveys of children in this village found a moderate level of active trachoma (18% follicular trachoma in children aged 1–9 years). After an initial census, adults with conjunctival scarring (grade S1b or worse, see below) were recruited as cases. An equal number of control subjects without scarring, frequency matched by ethnicity, were recruited. Individuals with trichiasis or previous eyelid surgery were excluded from this study.

Most of the cases in the TS study had relatively mild conjunctival scarring. Therefore, to characterize the full range of the scarring phenotype, a second case-control study was conducted in which all the cases were individuals with trachomatous trichiasis (TT), who generally had more severe conjunctival scarring. The cases in the TT study were recruited from patients undergoing trichiasis surgery in the Kilimanjaro Region. Control subjects were recruited from patients undergoing cataract or retinal detachment surgery at Kilimanjaro Christian Medical Centre.

### Clinical Assessment

All subjects were examined by an ophthalmologist (VH) using ×2.5 loupes and a bright torch. Examinations were carried out in a darkened tent or room. The 1981 World Health Organization trachoma grading system was used to grade conjunctival follicles and papillae, entropion, trichiasis, and corneal opacity.^11^ We developed a more detailed grading system for conjunctival scarring (Table 1 and Fig 1, available at http://aaojournal.org). A slit lamp was used to rule out subtle conjunctival scarring in normal controls.

### Confocal Microscopy Assessment

The IVCM examination of the upper tarsal conjunctiva was performed using the Heidelberg Retina Tomograph 3 in combination with the Rostock Corneal Module (Heidelberg Engineering GmbH, Dossenheim, Germany) using a previously described protocol.^10^ For the TS study, all IVCM assessments were done on the left eye, whereas for the TT study a mixture of right and left eyes were examined. Ten “volume” scans were taken from random locations across the tarsal conjunctiva, each of which consisted of 40 coronal scans taken in rapid succession at 2.1-μm intervals, starting at the conjunctival surface, moving from superficial to deep.

**Figure 2:**
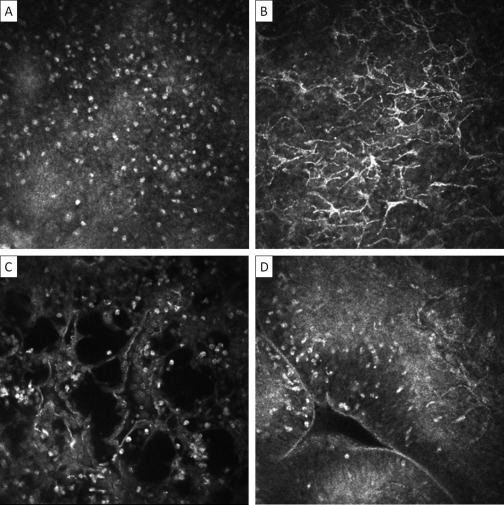
In vivo confocal microscopy grading system of inflammatory features. Images are 400×400 μm. **A,** Inflammatory infiltrate: seen as multiple bright white nuclei. The mean inflammatory cell density of 3 randomly selected volume scans is calculated. The individual scan with the highest density of cells from within the volume scan is used. **B,** Dendritiform cells: graded as present or absent. To be present, the mean number of DCs per volume scan needs to be ≥1. The largest number of DCs in any individual scan in a volume scan is used for measurement. A mean number of ≥1 is used to differentiate occasional DCs seen in scans of otherwise normal subjects. **C,** Tissue edema: seen as multiple black empty spaces. Graded as present or absent (present if seen in any volume scan). **D,** Papillae: seen as elevations with a central vascular network. Graded as present or absent (present if seen in any volume scan).

**Figure 3:**
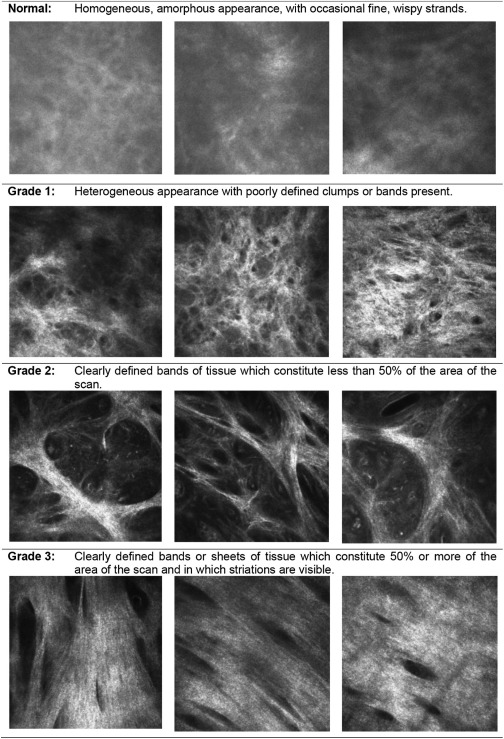
In vivo confocal microscopy grading system for conjunctival connective tissue organization. Images are 400×400 μm. If different grades of scarring are seen within a particular volume scan, then the highest grade is recorded. The connective tissue that is graded needs to be separate from that associated with the vascular tissue; if this is not possible, then the scan is considered ungradable.

The IVCM images were graded for inflammatory features (Fig 2) and the degree of subepithelial connective tissue organization (Fig 3).^10^ All IVCM grading was performed by a single observer (VH) who was masked to the clinical status of the patient, with the exception of the cell counts for the inflammatory infiltrate in the TS study, which was done by a single trained observer. For every subject, each volume scan was assessed and scored: 0 (normal), 1 (grade 1), 2 (grade 2), or 3 (grade 3) for the connective tissue organization grading. The overall connective tissue organization score for that subject was calculated by dividing the sum of these separate volume scan scores by the number of volume scans graded. Individuals with fewer than 3 gradable volume scans were excluded from the analysis. We have previously reported good interobserver agreement (intraclass coefficient of 0.88).^10^

### Sample Size and Data Analysis

These studies are part of a series of related studies on the pathogenesis of TS with the sample size calculated to encompass these other components. For example, the sample of 363 cases and 363 controls has >85% power to detect an association of a factor with an odds ratio (OR) of 2 when the factor is present in 10% of control subjects.

Data were entered into Access 2007 (Microsoft Corp, Redmond, WA) and analyzed using STATA 11.0 (StataCorp LP, College Station, TX). Logistic regression models were developed for clinical scarring to estimate ORs and 95% confidence intervals (CIs) for association with demographic and categoric IVCM characteristics. In view of the differences in ages between cases and controls in the TS study and the known association of TS with age, all ORs were age-adjusted. Linear regression (age-adjusted for the TS study) was used to estimate the strength of associations between clinical scarring and continuous IVCM variables (inflammatory infiltrate and connective tissue organization score). The TS study had a larger number of participants, so a more detailed analysis is also presented for this study. Multivariable logistic regression models (for categoric outcomes) and multivariable linear regression models (for continuous outcomes) were fitted to assess whether IVCM characteristics were independently associated with the presence of scarring, the level of scarring, and the level of clinical inflammation after adjusting for potential confounding factors. Likelihood ratio tests were used to assess the strength of association of factors with scarring. Tests for nonlinearity were conducted to assess whether fitting factors on a linear scale provided an adequate fit to the data. Tests for trend were used to examine the association of IVCM parameters with the ordered categories of clinical scarring and inflammation severity using the Wald test.

## Results

### Study Participants

The TS study village had an adult population of 3626, of whom 2418 (67%) were seen. Of the 1208 individuals not seen, the majority (58.9%) were absent during the census, despite 2 visits; 9.6% were temporarily resident elsewhere; and 4.1% refused examination. Of the 2418 participants seen, we excluded 36 (1.5%) because of the presence of trichiasis, previous eyelid surgery, or inability to give informed consent. Of the remaining 2382 participants, 862 (36.2%) had clinically apparent TS and 1520 (63.8%) did not have visible scarring. We recruited 363 TS cases and 363 controls. Demographic and clinical characteristics are shown in Table 2. Cases were significantly older than controls (*P* < 0.001), so subsequent analyses were adjusted for age. The majority of cases and controls were of Maasai ethnicity (77% of both groups) followed by Chagga ethnicity (11% of both groups).

We recruited 34 cases and 33 controls for the TT study (Table 3). There was no significant difference between the ages of TT cases and controls. The majority of the cases (85%) were Maasai, whereas the controls were more evenly divided between different ethnic groups (the largest groups were Chagga, 42%, and Pare, 24%).

### Analysis Based on Case-Control Status

The clinical scarring was mostly mild to moderate in the TS study and severe in the TT study (Tables 2 and 3). Most of the cases were clinically inflamed; in contrast, most controls were not. In the TT study most cases had severe clinical scarring (70% with S3), in contrast with the TS study (3.6% with S3).

In all participants, most of the cellularity detected by IVCM was found within the most superficial 20–30 μm of the conjunctiva, with cells not usually seen below this level. There were more inflammatory cells in cases than controls, significantly so in the larger TS study (Table 4). Cases were also more likely than controls to have dendritiform cells (DCs) and tissue edema present, but there was no association with the presence of papillae (Table 4).

The mean IVCM connective tissue organization score was higher in cases than in controls in both studies, but particularly in the TT study in which the cases had more severe clinical scarring (TT cases 2.29 vs. controls 0.59; *P* < 0.001; Table 4). The tissue organization score was similar in the control groups of both studies (TT study controls 0.59 vs. TS study controls 0.77). The IVCM connective tissue organization score was categorized into 3 groups (0–1, >1–2, and >2–3). In both studies, the majority of controls had a score of ≤1 (79% in the TS study and 92% in the TT study), compared with the cases (26% in the TS study and 8% in the TT study). Most of the cases in the TS study (63/81; 78%) with an IVCM score in the lowest 0–1 category had the mildest degree of clinical scarring, S1b. There were no controls in either study with the highest IVCM connective tissue organization category (2–3), which is characterized by clearly defined bands or sheets of tissue.

A multivariable logistic regression model of the TS study showed that after controlling for age and clinical inflammation, clinical scarring remained associated with the presence of DCs (OR 4.27, 95% CI, 1.39–13.09, *P=*0.008, Table 5, available at http://aaojournal.org). The corresponding adjusted association is even stronger for the TT study (OR 25.36, 95% CI, 1.69–380.00, *P =* 0.02). Tissue edema was not significantly associated with scarring after adjusting for age and clinical inflammation.

### Analysis Based on Clinical Scarring Grade (TS Study Only)

The IVCM inflammatory infiltrate, connective tissue organization score, presence of DCs, and presence of tissue edema all increased with the clinical scarring grade (Table 6, available at http://aaojournal.org). Regression models were used to analyze the association of each of these factors adjusting for age, sex, and the clinical inflammation grade. For modeling purposes, clinical scarring grades S2 and S3 were combined, because there were few individuals in clinical scarring grade S3 in the TS study. For each unit increase in the clinical scarring grade, there was an independent increase of 87 cells/mm^2^ in the inflammatory infiltrate (95% CI, 45–130; *P* < 0.001) and of 0.36 in the organization score (95% CI, 0.29–0.43; *P* < 0.001). Similarly, the OR for the presence of DCs increased by 1.68 (95% CI, 1.14–2.47, *P =* 0.008) for each increase in clinical scarring grade. The presence of tissue edema was not significantly associated with increasing clinical scarring grade (OR = 0.83, 95% CI, 0.43–1.61).

For reference, the mean connective tissue organization score for clinical scarring grade S3 in the TT study was 2.65 (95% CI, 2.36–2.94). Of these severely scarred cases, 13 of 16 (81%) were in the highest IVCM organization grade 2–3, and 3 of 16 (19%) were in the intermediate grade >1–2.

### Analysis Based on Clinical Inflammation Grade (TS Study Only)

The IVCM inflammatory infiltrate, connective tissue organization score, presence of DCs, and presence of tissue edema all tended to increase with the clinical inflammation grade (Table 7, available at http://aaojournal.org). Regression models were used to analyze the association of each of these factors, adjusting for age, sex, and clinical scarring grade, in a similar manner to that above. For each unit increase in the clinical inflammation grade, there was a small increase in the inflammatory infiltrate, but this was of only borderline statistical significance (*P* = 0.06). After adjusting for the other factors, the organization score no longer remained significantly associated with the level of clinical inflammation. The presence of DCs did remain associated with the level of clinical inflammation (*P*=0.03), especially for the highest grade of inflammation (OR 12.1, 95% CI, 2.1–69.2). With each increase in clinical inflammation grade, there was an increase of 5.12 in the OR for the presence of tissue edema (95% CI, 2.69–9.76, *P*<0.001).

## Discussion

In this study, IVCM was used to examine tissue changes in a large number of people with trachomatous conjunctival scarring and compared with controls. Previously developed grading systems were used to formally evaluate tissue morphology and cellular appearances.

Previous studies have shown that the cellular infiltrate is probably composed of inflammatory cells, such as neutrophils or lymphocytes, and is increased in patients with atopic keratoconjunctivitis.^8,12-15^ In vivo confocal microscopy assessment of the inflammatory cell density also has shown good correlation with assessment by brush cytology.^15^ Some attempt can be made to differentiate these cells on the basis of nuclear detail, for example, segmentation suggesting polymorphs, but definitive identification would require biopsy. We found that the inflammatory cell density increased with increasing clinical scores for both conjunctival inflammation and scarring. The association with scarring was greater than with inflammation and remained strongly significant even after adjusting for the level of clinical inflammation. This suggests that the increased cellularity may be more closely related to the scarring process than clinically visible “inflammation,” which is characterized by erythema and edema.

Dendritiform cells were found to be independently associated with trachomatous scarring. These cells have invariably been labeled as dendritic cells in previous studies of the ocular surface using IVCM, although there has not been definitive histologic confirmation of this finding.^7,16^ Dendritic cells play a central role in determining the type of immune response that develops to response to infection, which is of particular relevance in trachoma because the tissue damage is immune mediated.^17-21^ The view that dendritic cells are likely to play an important role in the design of any chlamydial vaccine gains some support from our study.^22^

Of note, most of the cells seen in these adults with TS were within the superficial 30 μm of the surface. This distribution suggests close interaction between these cells and the epithelium and lends support to the cellular paradigm of chlamydia pathogenesis. The cellular paradigm suggests that non-immune host cells, particularly epithelial cells, release proinflammatory cytokines and chemokines in response to chlamydial and other bacterial infection, which induces an inflammatory response leading to tissue damage.^3,23,24^ This is in contrast with the immunologic paradigm, which argues that the damage is primarily driven by an acquired cell-mediated immune response that is important for defense against infection but also leads to collateral tissue damage.^22^

Some attempt has been made at grading the density of conjunctival subepithelial connective tissue in relation to blebs after trabeculectomy surgery.^25^ However, no clear definitions were provided for this grading system, nor was there any evaluation of interobserver variation. In this article, we have applied the use of a grading system that has clear definitions and good interobserver agreement with the graders masked to the clinical findings.^10^ This study showed that scarring of the conjunctiva has a characteristic appearance on IVCM with well-defined bands or sheets of scarring seen. These probably represent collagen fibers. The IVCM organization score tended to show an increase with the clinical scarring grade. Most of the cases in the TT study had severe scarring, and most were also scored as being in the highest IVCM connective tissue organization grade, showing good agreement between clinical and IVCM assessment. Not all cases had an IVCM appearance suggestive of scarring, with some having a homogenous appearance similar to control subjects. However, most of these had very mild clinical scarring.

Some control subjects also had a more organized appearance of the connective tissue. This may represent limitations in the grading system or the quality of the images gained by the microscope. However, we have also performed tarsal conjunctival IVCM with a similar protocol on 30 healthy volunteer subjects from a variety of non-trachoma endemic areas (mean age 36.7 years, range 22–85 years). These scans were graded in a masked manner (mixed with scans from cases with scarring so that the grader, VH, did not know the clinical status), and the mean IVCM connective tissue organization score was 0.40 (95% CI, 0.27–0.54) with all individuals graded as being in the lowest IVCM organization grade. Although this may reflect differences based on ethnicity, an alternative explanation is that the confocal microscope is able to detect subclinical scarring. Many of the control subjects in both case-control studies presented were probably exposed to chlamydial infection as children. There is likely to be a spectrum of scarring severity seen in the population, some of which may not be visible clinically but can be detected with the confocal microscope. This may be represented by the 20% of clinically normal subjects in the TS study with an intermediate grade of IVCM organization. This is supported by the observation that in people from non-trachoma endemic areas, the mean IVCM organization score was lower than that of the controls in both case-control studies, and that the entire group had only the lowest grade of IVCM organization.

There are a number of limitations to these studies. In the TS study, cases tended to be older than controls. However, we adjusted for this in the analysis. The age distribution was similar between cases and controls in the TT study. Also, not all potential study participants were able to tolerate confocal microscopy. However, we think it is unlikely that any systematic bias was introduced that would have affected the results. We are currently comparing IVCM findings with paired histology specimens to further validate the interpretation of these observations. These studies were conducted in a district previously found to be endemic for trachoma, and results may differ in other areas/countries, for example, where levels of infection are hyperendemic. Although the cases and controls in the TS study were recruited from the same community, the controls in the TT study were from a mixture of urban and rural areas.

In conclusion, these studies have used IVCM to explore the cellular and tissue changes occurring in trachomatous conjunctival scarring and inflammation. A robust grading system has been used, and new insights have been gained into the pathologic mechanisms at work. We hope the grading system may be of relevance in the study of other immunofibrogenic diseases of the conjunctiva, such as mucous membrane pemphigoid and atopic keratoconjunctivitis.

## Figures and Tables

**Table 2:** Demographic and Clinical Characteristics of the Trachomatous Scarring Study Participants and Age-Adjusted Associations with Conjunctival Scarring

Parameter	Cases N = 363		Controls N = 363		Age-Adjusted Association with Scarring		
	n	(%)	n	(%)	OR	95% CI	*P* Value
*Demographic Characteristics*							
Sex							
Female	222	(61.2)	242	(66.7)	1.00	–	
Male	141	(38.8)	121	(33.3)	1.19	0.83–1.71	0.34
Age (year groups)					2.20⁎	1.94–2.50	<0.001
18–25	29	(8.0)	117	(32.2)			
25–35	53	(14.6)	114	(31.4)			
35–45	65	(17.9)	87	(24.0)			
45–55	81	(22.3)	28	(7.7)			
55–65	58	(16.0)	9	(2.5)			
> 65	77	(21.2)	8	(2.2)			
Age (yrs) [median, IQR]	49.7	36.7–62.7	29.2	22.2–39.2			
Formal education†							0.001
None	250	(69.4)	166	(46.1)	1.00	–	–
1–7 yrs	106	(29.4)	177	(49.2)	0.57	0.40–0.81	
> 7 yrs	4	(1.1)	17	(4.7)	0.25	0.07–0.88	
BMI‡							0.48
Underweight	55	(15.3)	30	(8.4)	1.39	0.79–2.44	
Normal	259	(72.0)	287	(80.2)	1.00	–	–
Overweight/obese	46	(12.8)	41	(11.5)	1.14	0.68–1.92	
*Clinical Characteristics*							
Scarring grade§							
S0	–	–	363	(100)			
S1a	0	0	–	–			
S1b	188	(51.8)	–	–			
S1c	128	(35.3)	−	−			
S2	34	(9.4)	–	–			
S3	13	(3.6)	−	−			
Papillary inflammation grade							
P0	81	(22.3)	346	(95.3)			
P1	189	(52.1)	17	(4.7)			
P2	85	(23.4)	0	(0.0)			
P3	8	(2.2)	0	(0.0)			
Follicles grade							
F0	358	(98.6)	358	(99.7)			
F1	2	(0.6)	1	(0.3)			
F2	1	(0.3)	0	(0.0)			
F3	2	(0.6)	0	(0.0)			

BMI = body mass index; CI = confidence interval; IQR = interquartile range; OR = odds ratio.

⁎ The result shown is the increase in the OR with each increase in age group category.

† Primary schooling in Tanzania is completed after 7 years.

‡ Categories from the National Institutes of Health.

§ All of the controls had no scarring (grade 0), and all of the cases had scarring grade ≥S1b.

**Table 3:** Demographic and Clinical Characteristics of the Trachomatous Trichiasis Study Participants and Associations with Conjunctival Scarring

	Cases N = 34		Controls N = 33		Association with Scarring		
Parameter	n	(%)	n	(%)	OR	95% CI	*P* Value
*Demographic Characteristics*							
Sex							
Female	23	(67.7)	15	(45.5)	1.00	–	
Male	11	(32.3)	18	(54.5)	2.61	0.93–7.34	0.06
Age (year groups)					1.62⁎	0.90–2.95	0.11
18–25	0	(0.0)	0	(0.0)			
25–35	0	(0.0)	0	(0.0)			
35–45	1	(6.9)	2	(6.1)			
45–55	1	(20.7)	7	(21.2)			
55–65	10	(20.7)	6	(18.2)			
> 65	22	(51.7)	18	(54.6)			
Age (yrs) [median, IQR]	68.5	61.0–80.0	70.0	53.1–78.1			
Formal education†							<0.001
None	28	(87.5)	4	(13.3)	1.00	–	–
1–7 yrs	4	(12.5)	18	(60.0)	0.02	0.00–0.12	
>7 yrs	0	(0.0)	8	(26.7)	0.02	0.00–0.18	
BMI	Data not collected						
*Clinical Characteristics*							
Scarring grade‡							
S0	–	–	33	(100)			
S1a	0	0	–	–			
S1b	0	0	–	–			
S1c	6	(17.7)	–	–			
S2	4	(11.8)	–	–			
S3	24	(70.6)	–	–			
Papillary inflammation grade							
P0	4	(11.8)	31	(93.9)			
P1	5	(14.7)	1	(3.0)			
P2	17	(50.0)	1	(3.0)			
P3	8	(23.5)	0	(0.0)			
Follicles grade							
F0	34	(100.0)	33	(100.0)			
F1	0	(0.0)	0	(0.0)			
F2	0	(0.0)	0	(0.0)			
F3	0	(0.0)	0	(0.0)			

BMI = body mass index; CI = confidence interval; IQR = interquartile range; OR = odds ratio.

⁎ The result shown is the increase in the OR with each increase in age group category.

† Primary schooling in Tanzania is completed after 7 years.

‡ All of the controls had no scarring (grade 0), and all of the cases had scarring grade ≥S1c.

**Table 4:** In vivo confocal microscopy findings from the Trachomatous Scaring and Trachomatous Trichiasis Studies and associations with conjunctival scarring

Parameter	Cases		Controls		Association with scarring†		
					OR	95%CI	*P* Value
	N = 328⁎		N = 319⁎				
**TS Study**							
Mean inflammatory infiltrate (cells/mm^2^) [mean, 95%CI]	875	832–919	674	640–707	*154*‡	*90–217*	*<0.001*
Dendritiform cells present, n(%)	50	(15.2)	7	(2.2)	4.10	1.71–9.85	0.002
Tissue edema present, n(%)	22	(6.7)	9	(2.8)	3.77	1.53–9.26	0.004
Papillae present, n(%)	110	(33.5)	129	(40.4)	1.14	0.78–1.66	0.49
	N = 314		N = 302				
Connective Tissue Organization grade, n(%)							<0.001
0–1	81	(25.8)	238	(78.8)	1.00	–	
1–2	178	(56.7)	64	(21.2)	6.2	4.1–9.4	
2–3	55	(17.5)	0	(0.0)	107.4§	14.2–813.7	
Mean connective tissue organization score (mean, 95%CI)							
	1.48	1.41–1.56	0.77	0.71–0.82	*0.59*‡	*0.48–0.69*	*<0.001*
**TT Study**							
	N = 28		N = 26				
Mean inflammatory infiltrate (cells/mm^2^) [mean, 95%CI]	1572	1174–1972	1252	1037–1467			0.11
Dendritiform cells present, n(%)	16	(61.5)	1	(3.6)	43.20	5.05–369.6	0.001
Tissue edema present, n(%)	10	(38.5)	0	(0.0)	16.9§	1.97–144.4	0.01
Papillae present, n(%)	0	(0.0)	3	(10.7)	0.33§	0.03–3.43	0.36
	N = 26		N = 25				
Connective Tissue Organization grade, n(%)							<0.001
0–1	2	(8.0)	24	(92.3)	1.00	–	
1–2	6	(24.0)	2	(7.7)	72.0	5.6–933.0	
2–3	17	(68.0)	0	(0.0)	204.0§	17.1–2434.9	
Mean connective tissue organization score (mean, 95%CI)							
	2.29	1.94–2.64	0.59	0.41–0.77			<0.001

OR = Odds ratio; CI = Confidence intervals; TS = Trachomatous Scarring; TT = Trachomatous Trichiasis.

⁎ There are different N numbers as not all subjects had images which could be graded.

† Age-adjusted for the TS Study.

‡ Age-adjusted difference between cases and controls.

§ If a cell contained 0 then a participant was moved up a category in order to be able to generate a representative odds ratio.
