# Supplementary material for: In Vivo Confocal Microscopy in Scarring Trachoma
Source: Ophthalmology. 2011 Nov;118(11-2):2138–46. doi: 10.1016/j.ophtha.2011.04.014 (PMC3267045; doi:10.1016/j.ophtha.2011.04.014)
Supplement: Table 1 [file mmc1.pdf]

**Table 1:** Clinical Scarring Grading System for the tarsal conjunctiva.

| Grade | | Definition* |
|---|---|---|
| S1 | | Scarring occupying <⅓ of the upper lid |
| | S1a | One or more pinpoint scars and/or a single line of scarring less than 2mm in length† |
| | S1b | Multiples lines of scarring less than 2mm in length |
| | S1c | One or more lines/patches of scarring each 2mm or more in length/maximal dimension |
| S2 | | Patches of scarring occupying in surface area ≥ ⅓ but < ⅔ of the upper lid |
| S3 | | Patches of scarring occupying in surface area ≥ ⅔ of the upper lid |

* ”upper lid” refers to zones 2 and 3 of the everted lid.[29]

† 2mm was chosen as this is the approximate width of the lower lid margin, which is readily available for comparison.
