# Supplementary material for: In Vivo Confocal Microscopy in Scarring Trachoma
Source: Ophthalmology. 2011 Nov;118(11-2):2138–46. doi: 10.1016/j.ophtha.2011.04.014 (PMC3267045; doi:10.1016/j.ophtha.2011.04.014)
Supplement: Table 5 [file mmc2.pdf]

Table 5: Multivariable logistic regression model for the presence of conjunctival scarring (Trachomatous Scarring Study subjects only)

Risk factor	Odds Ratio	95% CI	p-value
Age group*	2.26	1.86-2.75	<0.001
Presence of any clinical inflammation	72.45	38-138	<0.001
Dendritiform cells present	4.27	1.39-13.04	0.008

* The result shows the increase in the OR with each increasing age group (10 year) category.

CI = Confidence intervals
