# Supplementary material for: In Vivo Confocal Microscopy in Scarring Trachoma
Source: Ophthalmology. 2011 Nov;118(11-2):2138–46. doi: 10.1016/j.ophtha.2011.04.014 (PMC3267045; doi:10.1016/j.ophtha.2011.04.014)
Supplement: Table 6 [file mmc3.pdf]

Table 6: *In vivo* confocal microscopy parameters by Clinical Scarring Grade (Trachomatous Scarring study subjects only).

Parameter	Clinical Scarring Grade										Test for trend*
	S0		S1b		S1c		S2		S3		
Mean inflammatory infiltrate (cells/mm2) [mean (95%CI)]	674	(640-707)	783	(730-835)	964	(898-1031)	1028	(902-1155)	1045	(844-1245)	<0.001
Mean connective tissue organization score [mean (95%CI)]	0.77	(0.72-0.82)	1.30	(1.21-1.39)	1.60	(1.47-1.73)	2.06	(1.83-2.29)	1.54	(1.21-1.88)	<0.001
Dendritiform cells present [n (%)]	7	(2.19)	18	(10.34)	20	(18.35)	6	(18.75)	6	(46.15)	0.008
Tissue edema present [n (%)]	9	(2.82)	6	(3.45)	12	(11.01)	2	(6.25)	2	(15.38)	0.58

* Adjusted for age, sex and Clinical Inflammation Grade

CI = Confidence interval
