# Supplementary material for: In Vivo Confocal Microscopy in Scarring Trachoma
Source: Ophthalmology. 2011 Nov;118(11-2):2138–46. doi: 10.1016/j.ophtha.2011.04.014 (PMC3267045; doi:10.1016/j.ophtha.2011.04.014)
Supplement: Table 7 [file mmc4.pdf]

Table 7: *In vivo* confocal microscopy parameters by Clinical Inflammation Grade (Trachomatous Scarring Study subjects only).

Parameter	Clinical Inflammation Grade								Test for trend*
	P0		P1		P2		P3		
IVCM inflammatory infiltrate score (cells/mm2) [mean (95%CI)]	693	(657-730)	850	(799-902)	983	(902-1063)	974	(724-1224)	0.06
IVCM connective tissue organization score [mean (95%CI)]	0.91	(0.85-0.97)	1.42	(1.33-1.52)	1.51	(1.36-1.66)	1.48	(0.97-1.98)	0.23
Dendritiform cells present [n (%)]	18	(4.8)	20	(10.9)	14	(17.8)	5	(62.5)	0.03
Tissue edema present [n (%)]	9	(2.4)	4	(2.2)	13	(16.7)	5	(62.5)	<0.001

* Adjusted for age, sex and Clinical Scarring Grade

CI = Confidence interval
