# Supplementary material for: In Vivo Confocal Microscopy in Scarring Trachoma
Source: Ophthalmology. 2011 Nov;118(11-2):2138–46. doi: 10.1016/j.ophtha.2011.04.014 (PMC3267045; doi:10.1016/j.ophtha.2011.04.014)
Supplement: Figure 1 [file mmc5.pdf]

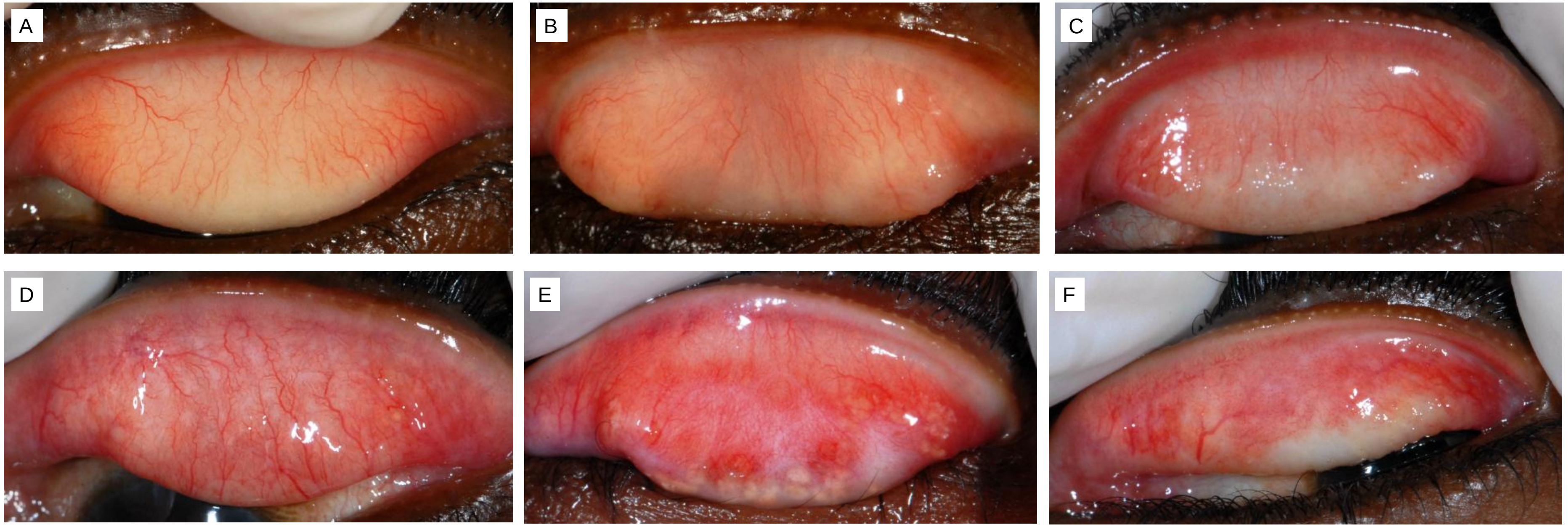

Figure 1. Clinical grading system for trachomatous conjunctival scarring. **A,** Normal. **B,** Grade S1a. **C,** Grade S1b. **D,** Grade S1c. **E,** Grade S2. **F,** Grade S3.
